# A bi-level framework for real-time crash risk forecasting using artificial intelligence-based video analytics

**DOI:** 10.1038/s41598-024-54391-4

**Published:** 2024-02-19

**Authors:** Fizza Hussain, Yasir Ali, Yuefeng Li, Md Mazharul Haque

**Affiliations:** 1 School of Civil and Environmental Engineering, Faculty of Engineering, Queensland University of Technology, Brisbane, 4001 Australia; 2 School of Architecture, Building, Civil Engineering, Loughborough University, Leicestershire, LE11 3TU UK; 3 School of Computer Science, Faculty of Science, Queensland University of Technology, Brisbane, 4001 Australia

**Keywords:** Civil engineering, Statistics

## Abstract

This study proposes a bi-level framework for real-time crash risk forecasting (RTCF) for signalised intersections, leveraging the temporal dependency among crash risks of contiguous time slices. At the first level of RTCF, a non-stationary generalised extreme value (GEV) model is developed to estimate the rear-end crash risk in real time (i.e., at a signal cycle level). Artificial intelligence techniques, like YOLO and DeepSort were used to extract traffic conflicts and time-varying covariates from traffic movement videos at three signalised intersections in Queensland, Australia. The estimated crash frequency from the non-stationary GEV model is compared against the historical crashes for the study locations (serving as ground truth), and the results indicate a close match between the estimated and observed crashes. Notably, the estimated mean crashes lie within the confidence intervals of observed crashes, further demonstrating the accuracy of the extreme value model. At the second level of RTCF, the estimated signal cycle crash risk is fed to a recurrent neural network to predict the crash risk of the subsequent signal cycles. Results reveal that the model can reasonably estimate crash risk for the next 20–25 min. The RTCF framework provides new pathways for proactive safety management at signalised intersections.

## Introduction

Conventional safety assessment at signalised intersections follows a reactive approach, whereby safety is often assessed after crashes have already occurred. This approach involves an ethical dilemma and limits the efforts to investigate safety because it requires an unusually large number of crashes. In addition, the police-recorded crash data does not provide sufficient information about the precursors of a crash. As such, proactive safety assessment based on traffic conflicts is gaining attention because it can overcome the shortcomings of crash-based (or conventional) analyses and drastically reduce the time required for safety assessments.

The foundation of proactive safety assessment rests on the notion of using traffic conflicts instead of crashes that can provide insights into the crash occurrence mechanism. Along this line, Hydén^1^ proposed a safety pyramid, enlisting a hierarchy of traffic events in a continuum framework. According to Hyden’s pyramid, crashes can be linked with traffic conflicts, and for this purpose, Extreme Value Theory (EVT) has been frequently used. EVT is a non-crash-based approach that can extrapolate crash risk from frequently observable events (traffic conflicts) to rare events (crashes)^2^. EVT also provides a single dimension for measuring the severity of extreme traffic conflicts and identifying crashes, perfectly aligning within the classic safety hierarchy framework (see Hydén’s pyramid^1^) and abandoning the assumption of fixed crash-to-surrogate ratio^3^. Motivated by these unique characteristics of EVT, this study applies EVT to estimate and understand the crash risk in real-time.

Real-time safety assessment is often difficult to perform using conventional statistical models and historical crash data. Whilst existing studies on real-time crash prediction demonstrated promising results in predicting the occurrence/counts of crashes^4^, most of the studies have framed crash prediction as a binary classification problem where a discrete outcome of either “crash” or “no crash” is assigned to a set of traffic conditions rather than quantifying the associated level of riskiness. Overcoming this issue, a recent study applied the extreme value theory (EVT) approach to estimate the real-time safety of signalised intersections^5^. Their study used a non-stationary Bayesian Generalised Extreme Value model to estimate the distribution of each signal cycle from which safe and risky signal cycles were identified. This approach correlates crashes with traffic conditions (proxied by several metrics, such as traffic flow or volume, shockwave speed, shockwave area, platoon ratio and others), and it is expected that a future signal cycle with similar traffic conditions shall exhibit a crash risk close to one estimated in the past. Assessing the crash risk of future signal cycles by examining the pattern of crash risk that forms over time has not been explored. This approach would not only help forecast the crash risk of a site at a micro level but also proactively identify the spatiotemporal status of signalised intersections where safety is gradually deteriorating over time.

Motivated by this research gap, this study presents a bi-level framework for real-time crash risk forecasting (RTCF) for signalised intersections. More specifically, RTCF utilises extreme value theory (EVT) for estimating crash risk at a micro level whereas recurrent neural networks facilitate the forecasting of such crash risk in real-time for future time slices. This study, for the first time, estimates the crash risk of signalised intersections using machine learning-based time-series modelling. Whilst EVT assists in establishing the link between conflict extremes and crashes at the micro level, like a signal cycle, the time-series forecasting capability of recurrent neural networks has been leveraged to forecast the crash risk based on past and present crash risk. An automated covariate extraction algorithm for the signal cycle, proposed by Ali et al.^6^, is extended to calculate the covariates for rear-end conflicts automatically. This algorithm uses data fusion principles for combining three data sources.

The rest of the paper begins with summarising the literature review of relevant studies followed by study methodology and data collection. Modelling results are then presented and discussed in detail, and finally, study findings are summarised along with an outlook for future research.

## Literature review

This section briefly summarises the studies on real-time safety assessment that have utilised extreme value theory and machine learning techniques to provide a background of the research problem addressed by this study. Providing a comprehensive review of traffic conflict techniques is beyond the scope of this study, and interested readers are referred to earlier studies^7–10^.

### Extreme value theory for crash risk estimation

In the last decade, extreme value theory (EVT) has been frequently used to estimate crashes from traffic conflicts. In one of the early attempts, Songchitruksa and Tarko^2^ employed a block maxima (BM) approach to sample traffic conflicts obtained from video footage. This study showed promising results in estimating aggregated crashes for *n*-years utilising traffic conflicts. In another study, Zheng et al.^3^ compared BM and peak over threshold (POT) approaches for modelling freeway lane change crashes utilising the conflicts obtained from video footage. Although relatively high prediction error was reported whilst comparing the number of observed crashes with estimated crashes, their study confirmed that crashes could be estimated from conflicts and POT outperformed BM. Since then, many studies with similar and different objectives have applied EVT to estimate crashes, and a comprehensive review can be found in Ali et al.^10^.

Recognising extreme value theory (EVT)’s capability in estimating crashes, the real-time crash risk was assessed using EVT in a recent study^5^. This study developed a Bayesian hierarchical generalised extreme value model, which is used to estimate separate GEV distributions for each signal cycle from which safe and risky signal cycles were identified. Different from Zheng and Sayed^5^, Fu and Sayed^11^ developed a dynamic extreme value model, allowing parameters to be time-varying. The dynamic model was found to better identify signal cycles with positive crash risk or return level compared to Zheng and Sayed^5^’s model.

Although recent studies have used extreme value theory (EVT) models to evaluate real-time safety, these studies only estimated the crash risk for current time periods (e.g., the current signal cycle for a signalised intersection). By the time the traffic data of these cycles are analysed and processed, they are not current anymore. Therefore, these risk assessment models can also be argued to be passive. A “real-time” safety assessment should be capable of estimating the crash risk of future time periods, which has by and large not been explored yet, thus motivating the present study.

### Machine learning for real-time crash risk prediction

Over the years, machine learning has been extensively applied for crash prediction. This subsection briefly summarises the studies attempting to predict real-time crashes using machine learning techniques. Yuan et al.^12^ estimated real-time crash risk by considering time series dependency with a long short-term memory (LSTM) recurrent neural network algorithm. The developed model showed better accuracy in predicting crash occurrence using loop detector data than a conditional logistic model. Li et al.^13^ developed a hybrid of LSTM and convolutional neural network (CNN) to predict crashes on arterials. The CNN model is used to extract salient features from local raw data, whereas LSTM is used to capture long-term dependency. In another study, Cai et al.^14^ employed a deep convolutional generative adversarial network model to generate crash data and overcome the data imbalance issue. Using loop detector data from expressways, this machine learning model provided the best prediction accuracy and outperformed competing models like logistic regression, support vector machine, artificial neural network, and convolutional neural network. Whilst these studies predicted aggregated crashes and assessed safety in real time, estimating crash risk at the micro level is often overlooked, hindering the understanding of crash-prone conditions as reported in earlier studies^15–18^.

Notably, all these studies have developed crash-based models for predicting crash occurrence as a function of traffic conditions, weather factors and roadway geometric characteristics. However, the existing literature is devoid of studies that have applied machine learning techniques to estimate the crash risk of future time periods using traffic conflicts. To this end, the way forward could be to consider the temporal correlation of crash risk of past and present time periods to estimate the crash risk of future time periods. However, the notion of time-series forecasting of crash risk of future signal cycles for signalised intersections remains largely unexplored, perhaps due to the unavailability of crash risk at a signal cycle level. Understanding such crash risk for future signal cycles in advance will assist in devising risk-responsive countermeasures, which can minimise further crash risk propagation.

To summarise, the state-of-the-art real-time safety assessment either relies on historical crash data or lacks the ability to estimate the crash risk of future time periods. This study aims to address these limitations by developing a real-time crash risk forecasting framework (RTCF) by applying a hybrid of extreme value theory and machine learning (recurrent neural network).

## Methodology

The presence of temporal correlation in crash risks has been acknowledged in literature^19,20^, which enables the application of time-series prediction techniques such as recurrent neural networks. In the context of signalised intersections, this study extends the notion of temporal dependency for the crash risk of contiguous signal cycles. However, one significant difference between the existing studies and this study is that earlier studies have framed crash risk estimation as a classification problem^12,13,21^, whereas this study aims to quantify the crash risk probability (a continuous value between 0 and 1) in an autoregressive manner. In other words, it is assumed that the crash risk of future signal cycles can be predicted using the crash risk of current and past signal cycles as they form a time series. This approach to future crash risk prediction is distinctively different from existing studies on traffic safety.

**Figure 1:**
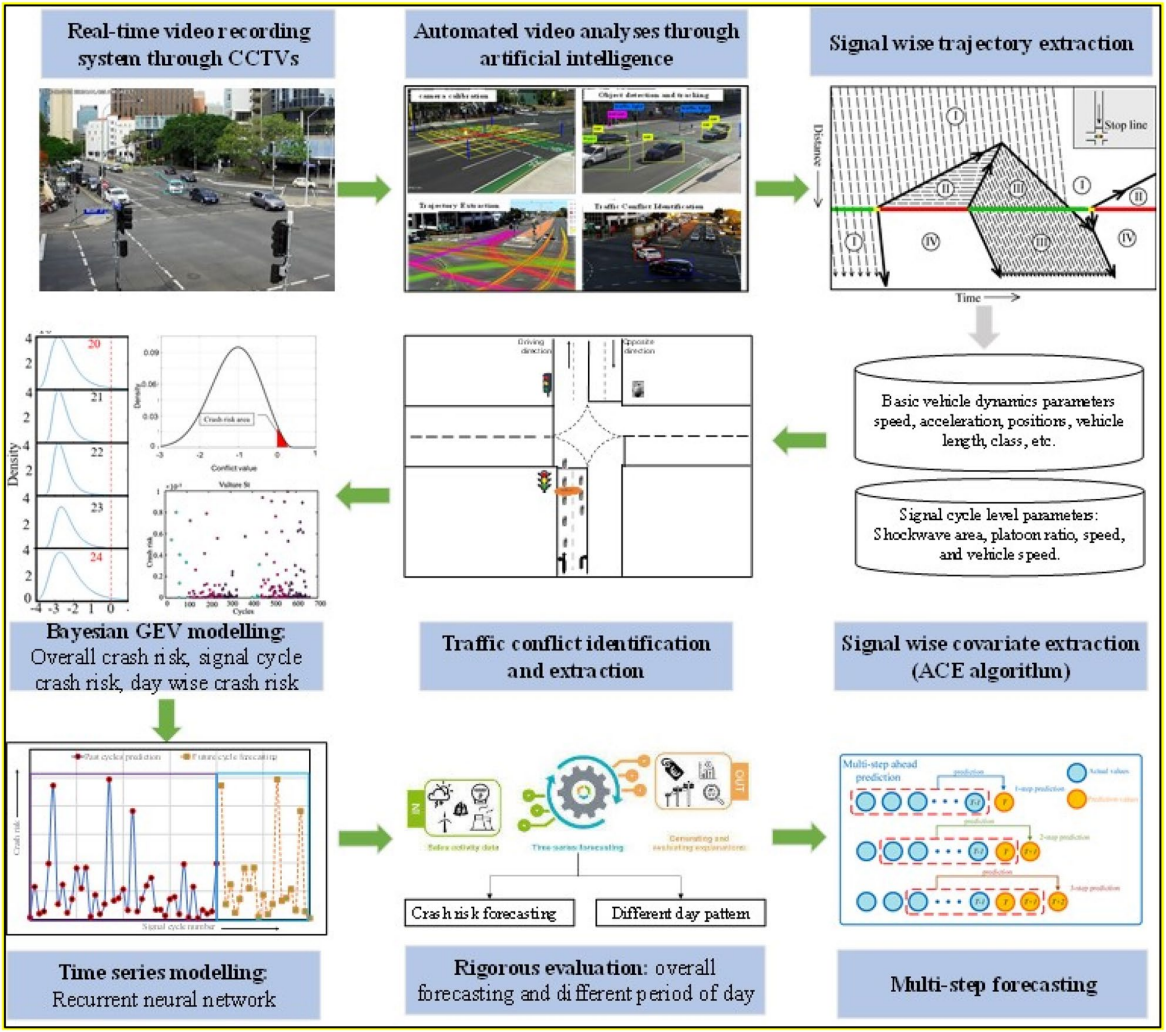
Schematic of the overall study.

As such, the first and foremost step is to obtain the crash risk probabilities of past signal cycles (time slices) from which the crash risk of future signal cycles can be predicted. To this end, this study proposes a bi-level real-time crash risk (RTCF) modelling framework, leveraging the combined benefits of extreme value theory and recurrent neural networks (RNNs). The overall study framework can be seen in Fig. 1. A Bayesian non-stationary generalised extreme value (GEV) model is developed at the first level, allowing the estimation of crash risk at the signal cycle level using rear-end traffic conflicts extracted from video footage. Although the proposed framework is generic and can be applied to any crash type, this study deals with rear-end crashes at signalised intersections for the following reasons. First, signalised intersections pose a significant risk to traversing vehicles due to the possible dilemma of stopping or crossing when a traffic light changes from green to red, thereby increasing the probability of rear-end or angle crashes at intersections. For instance, more than 3500 rear-end collisions occurred on Queensland roads in 2022, and approximately one-quarter of rear-end crashes led to serious injuries or fatalities, highlighting the frequency and severity of rear-end crashes^22,23^. Second, the frequency of rear-end conflicts is higher than other conflicts (e.g., vehicle–pedestrian), thereby providing a reasonable sample size for model estimation. To characterise time-varying crash risk, several covariates are computed using an algorithm based on the data fusion principle. To test the efficacy of the non-stationary Bayesian GEV model, the output of this model (i.e., estimated crashes) is first compared with historical crash records. Next, the crash risks of signal cycles estimated by the model are fed as input to a recurrent neural network model to predict the crash risk of future signal cycles. The predictions of the recurrent neural model are compared with the crash risk estimated by the extreme value theory model. Ensuing subsections detail each aspect of the RTCF framework.

### Extreme value theory model

The foundation of RTCF rests on extreme value theory (EVT), which has the capability to link traffic conflicts with crashes, thereby facilitating proactive safety management. To this end, a block maxima approach, which corresponds to a generalised extreme value (GEV) distribution, is adopted in this study. For this purpose, a signal cycle, which serves as a natural block for signalised intersections, is selected as a block. Assume that ${z}_{1},{z}_{2},{z}_{3},\dots {z}_{n}$ be a sequence of independent variables with a common distribution function and ${M}_{n}={{max(z}_{1},z}_{2},{z}_{3},\dots {z}_{n})$ yields the block maximum of *n* values. Whilst the proposed framework is generic, this study applies it to rear-end conflicts (characterised by modified time-to-collision). When n → ∞, the maximum of conflict values leads to a GEV distribution^3^ expressed as

1 $$G\left(z\right)=exp\left(-{\left[1+\xi \left(\frac{z-\mu }{\sigma }\right)\right]}^{-1/\xi }\right),$$

where − ∞ < μ < ∞ corresponds to the location parameter, σ > 0 represents the scale parameter, and − ∞ < ξ < ∞ denotes the shape parameter. From the fitted GEV distribution, the rear-end crash risk can be calculated as

2 $${RC}_{i}=Pr\left({z}_{i}>0\right)=1-{G}_{i}\left(0\right)=\left(\begin{array}{c}\begin{array}{cc}1-\mathit{exp}\left(-{\left[1-{\xi }_{i}\frac{{\mu }_{i}}{{\sigma }_{i}}\right]}^{-\frac{1}{\xi }}\right),& for \xi \end{array}\ne 0\\ \begin{array}{cc}1-\mathit{exp}\left[-exp\left(\frac{{\mu }_{i}}{{\sigma }_{i}}\right)\right],& for \end{array}\xi =0\end{array}\right),$$

where ${RC}_{i}$ represents the risk of a crash in signal cycle *i* (*i* = 632, 531, and 617 for Appleby, Beaudesert, and Logan intersections, respectively)*,* and $G(\cdot )$ is the fitted GEV distribution. The risk of a crash is nonnegative, with a value of zero indicating no crash risk for a given cycle (or a safe cycle) and a value greater than zero indicating a positive crash risk in a given signal cycle.

The block maxima model, as shown in Eq. (1), is applied to sample and model rear-end extremes. Ensuring the scale parameter to be positive, this study parameterises scale parameter as $GEV(\mu ,\phi ,\xi )$, where $\phi =log\sigma$. Let ${z}_{i}$ be the ${i}^{th}$ cycle maximum for a given site and $i=\mathrm{1,2},\dots {n}_{j}.$ As ${z}_{i}$ indicates the maximum value of a traffic conflict measure for cycle *i* at a site, a GEV distribution, which is site dependent, can be obtained as

3 $$G\left({z}_{i}<z|{\mu }_{i},{\phi }_{i},{\xi }_{i}\right)=exp\left(-{\left[1+{\xi }_{i}\left(\frac{z-{\mu }_{i}}{exp({\phi }_{i})}\right)\right]}^{-1/{\xi }_{i}}\right).$$

Rear-end conflicts are affected by several time-varying factors, such as traffic conditions. As such, not accounting for them during model estimation may lead to a time-varying unobserved heterogeneity issue, which has been reported to significantly affect model performance [2]. To circumvent this issue, several covariates are included in the GEV model parameters (except for the shape parameter that is difficult to estimate precisely, as noted by Coles^24^), and thus non-stationary models are developed. An identity link function has been adopted, which is a common way of including covariates. Mathematically, GEV parameters can be parameterised as

4 $$\left(\begin{array}{c}{\mu }_{{\text{i}}}={\alpha }_{\upmu 0}+{\alpha }_{\upmu 1}X+{\varepsilon }_{\upmu }\\ {\phi }_{{\text{i}}}={\alpha }_{\phi 0}+{\alpha }_{\phi 1}Y+{\varepsilon }_{\phi }\\ {\xi }_{{\text{i}}}={\alpha }_{\xi 0}+{\varepsilon }_{\xi }\end{array}\right),$$

where ${\alpha }_{\upmu 0}$, ${\alpha }_{\phi 0}$, and ${\alpha }_{\xi 0}$ are intercept terms corresponding to three model parameters, ${\alpha }_{\mu 1}$ and ${\alpha }_{\phi 1}$ are respectively parameter estimates for the covariate vectors ***X*** and ***Y***, and ${\varepsilon }_{\mu }$, ${\varepsilon }_{\phi }$, and ${\varepsilon }_{\xi }$ are random error terms. Note that random terms in Eq. (4) indicate between-site variances, which remain constant for extremes at the same site but vary between different sites.

A Bayesian estimation procedure is applied to estimate the model parameters shown in Eq. (4), which is suitable for obtaining posterior distributions and characterising the latent process by specifying priors for model parameters. Uninformative priors are assigned for all model parameters due to a lack of information on how generalised extreme value parameters vary. Priors are assumed to follow a normal distribution with mean zero and large variance, $N(0,{10}^{6})$. It has been reported in the literature that improperly defined priors for the shape parameter can potentially lead to convergence issues^5^. To address this issue, prior information from some previous studies^2,3^ is used where the shape parameter is reported between the range of (− 1.0,1.0). As such, the prior for the shape parameter is set as ${\alpha }_{\xi }\sim unif(-\mathrm{1.0,1.0})$. Further, the posterior distributions of model parameters are obtained using Markov Chain Monte Carlo simulation, where Gibbs sampling is used.

Several Bayesian generalised extreme value distribution models are developed using different combinations of covariates. The deviance information criterion (DIC) is the most commonly used performance measure in the Bayesian framework^25^, which can be expressed as

5 $$DIC= \overline{D }+{p}_{D}$$

where $\overline{{\text{D}} }$ is the posterior mean deviance that measures the model fitting and ${p}_{D}$ is the effective number of parameters in the model. In general, a smaller value of DIC indicates a better model fit. From the suite of developed models, the best model is selected using the smallest DIC value.

### Recurrent neural network model

**Figure 2:**
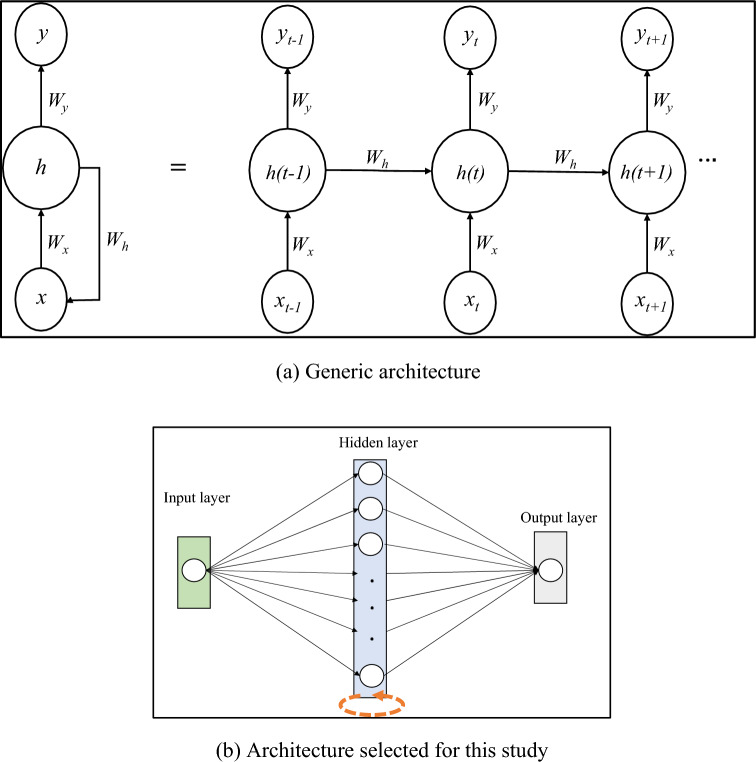
Recurrent neural network model architecture.

Recurrent neural networks (RNNs) are a specialised type of neural networks for modelling time-series or sequenced data that have serial dependency among observations. Unlike traditional feed-forward neural networks, these networks can memorise the hidden state of the network. This property enables them to learn the pattern among observations to predict the next value in the sequence. Mathematically, the principle of the operation of RNNs can be expressed by Eqs. (6) and (7)^26^, and the basic model architecture can be seen in Fig. 2.

6 $$h\left(t\right)={f}_{H}\left({W}_{x}x\left(t\right)+{W}_{h}h\left(t-1\right)\right),$$

7 $$y\left(t\right)={f}_{o}\left({W}_{y}h\left(t\right)\right),$$

where *x*(*t*) is the input (here, crash risk) at time *t*, *y*(*t*) is the output at time *t* (which is effectively the crash risk at time *t* + 1), *h*(*t*) is the hidden state of the network at time *t*, ${f}_{o}$ and ${f}_{H}$ are the hidden layer and output layer activation function, respectively, and ${W}_{x}$, ${W}_{h}$, and ${W}_{y}$ are the weight matrices of connections.

In this study, recurrent neural network (RNN) has been used to capture the temporal correlation among crashes and subsequently forecast the crash risk for future signal cycles. Since this study focusses on forecasting future crash risk by learning its temporal pattern rather than determining whether a crash will occur or not (classification), RNN is suited for this study. For each signalised intersection, video data of traffic movements were recorded for a period of 4 weekdays; however, the data was collected only during daylight hours (6 am–6 pm). Therefore, to feed the data to the RNN model, the 4 days of data of a given intersection have not been combined since it is not possible to place the first data point (here, crash risk of a signal cycle) of a day next to that the last data point of the next day because of a difference of 12 h. As such, the formed time series will be incorrect and violate the assumption of regularly spaced time intervals. Similarly, the data of different intersections is also not combined as the days of recording are not the same across intersections. Moreover, even if the data were to be collected on the same days for the three intersections, it is ideal to feed the data of every intersection separately to predict the real-time crash risk.

Several vanilla recurrent neural network (RNN) models without any stacking on neural networks were developed using different combinations of activation functions, optimisers, number of layers and number of neurons, as shown in Table 1. Note that these models were developed using Python’s *Keras* package. Moreover, long short-term memory (LSTM) networks were also tested but were not found to perform better than RNNs (further discussion on this can be found in Section VI***). Mean absolute error and relative absolute error (RAE) are used as the error metrics to evaluate the performance of candidate models. The best model in terms of the lowest RAE (and MAE) has a two-layered architecture: one recurrent layer composed of 50 neurons, whilst the second layer is a fully connected output layer. Rectified linear unit (ReLu) is used as the activation function, whereas Adam optimiser has been used for the back-propagation process. Additionally, for the RNN model, input sequence length is an important parameter that determines the number of data points to be fed to the RNN model to predict the next data point(s) of the sequence. For the final model, the sequence length of 12 gives the best prediction results.

**Table 1:** Tested hyperparameters and selected hyperparameters for the best model.

Model hyperparameter	Tested	Selected
Activation function	ReLU and Sigmoid	ReLU
Optimiser	Stochastic Gradient Descent and Adam	Adam
Number of neurons	10, 50, and 100	50
Sequence length	6, 12, and 24	12
Architecture	RNN and LSTM	RNN

## Data and pre-processing

### Video data collection and processing

**Figure 3:**
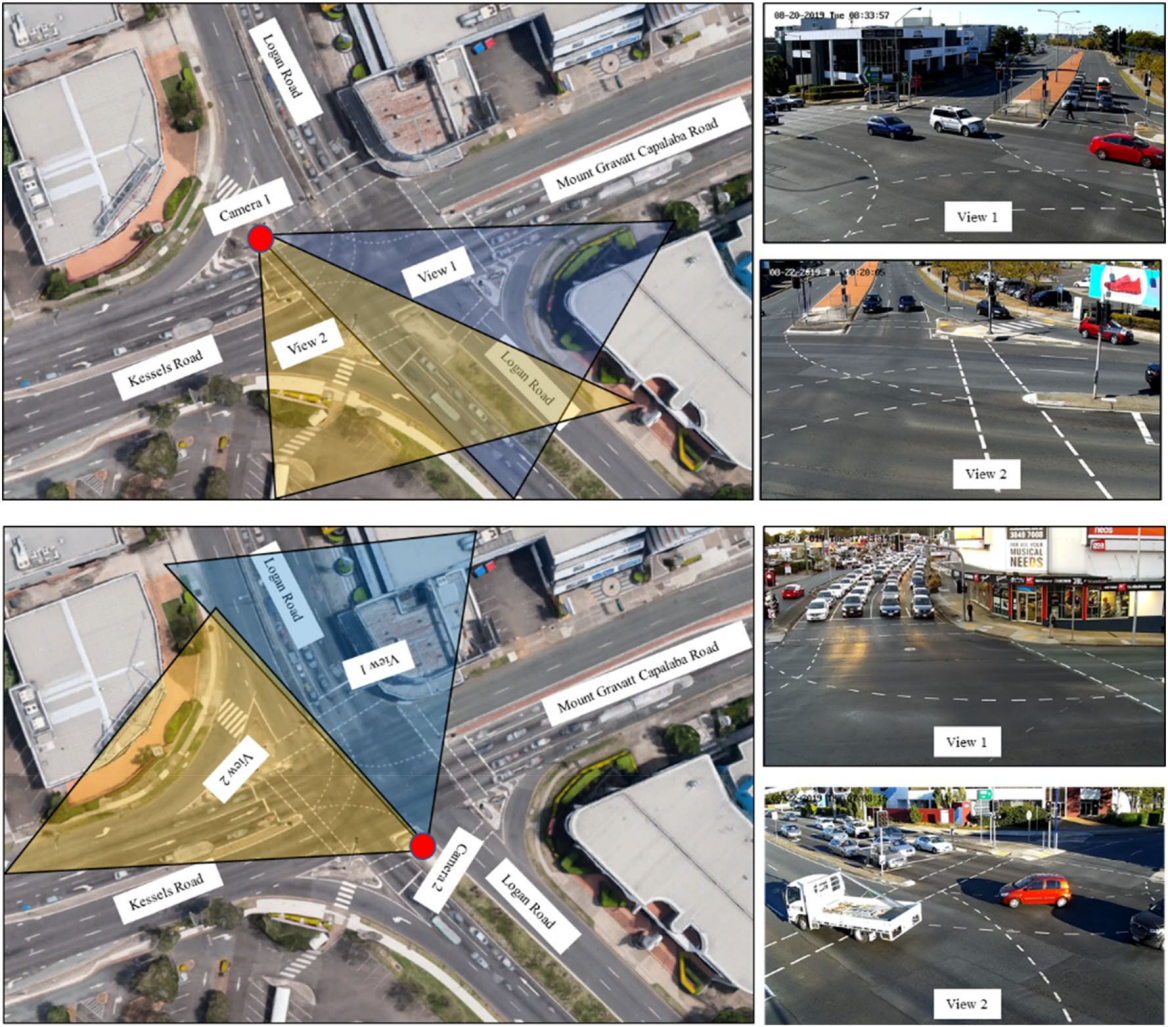
Camera positions and views at the Logan Rd–Kessel Rd intersection.

The proposed framework for real-time crash risk estimation (RTCF) is applied to three four-legged signalised intersections in Southeast Queensland, Queensland, Australia. Cameras were placed on a 6.5 m high pole near each intersection to record videos. Recognising the large area of an intersection, which could not be captured through a single camera, two cameras were used to capture the view of all the approaches of each intersection (Fig. 3). For all intersections, data were collected on 4 weekdays (Tuesday–Friday) and in the daytime only (6 am–6 pm).

**Figure 4:**
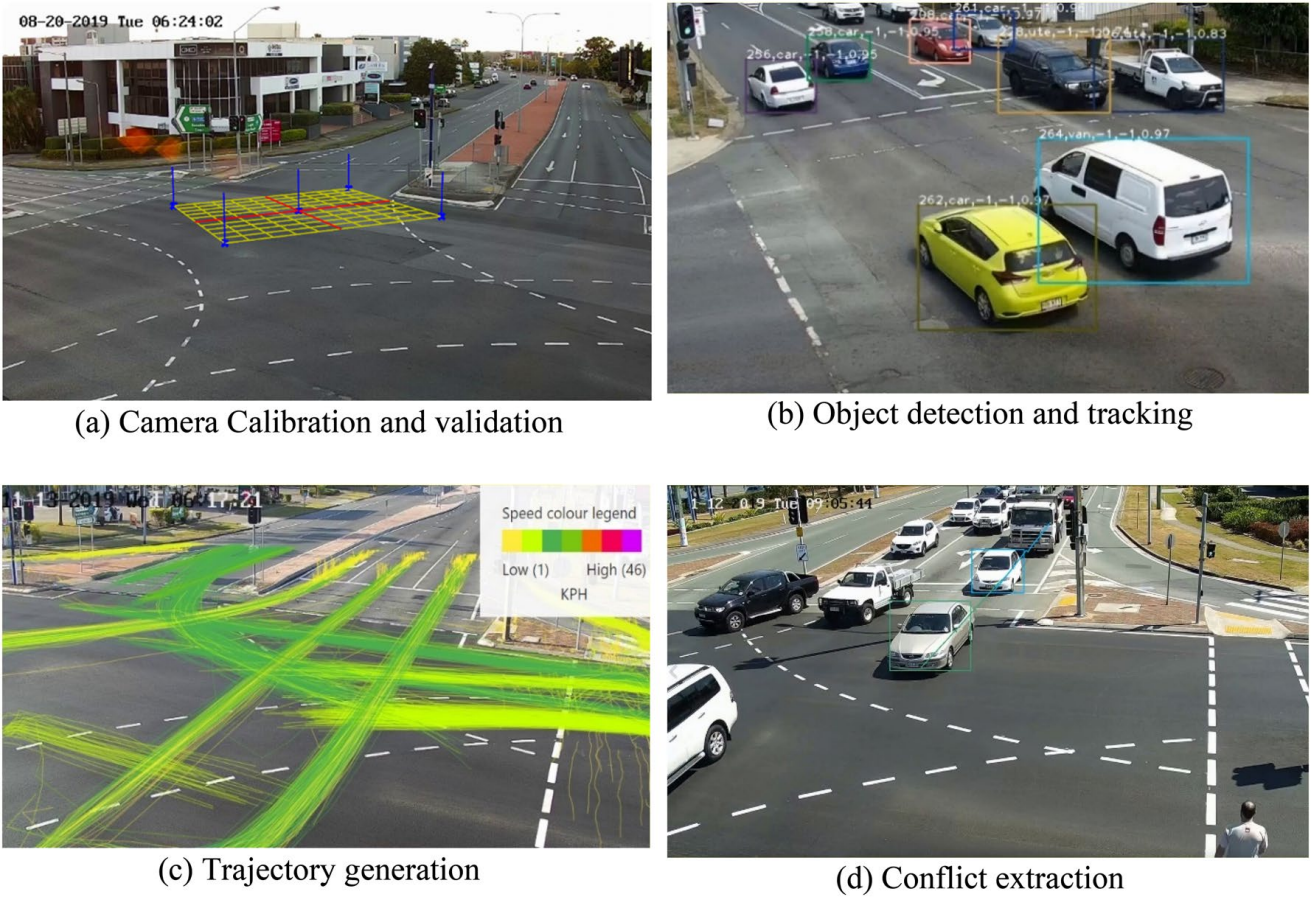
Traffic conflict identification process using artificial intelligence-based video analytics.

An artificial intelligence-based automated video analysis platform was used to process video footage. Figure 4 describes the key steps in video data processing, which include camera calibration, object detection and tracking, trajectory extraction, and conflict identification. Camera calibration is necessary for tracking vehicles in the camera image and translating these positions into the real world. Whilst a detailed description of video processing and its schematics are omitted from this paper, interested readers are referred to our earlier studies^27,28^. Succinctly, the YOLO algorithm was used for object detection in a traffic scene^29^, whereas the DeepSORT algorithm tracked the movement of the detected objects (both motorised and non-motorised). The trajectories obtained from automated analysis were meticulously examined and corrected for any measurement/calibration error. A trajectory overlapping technique identified safety–critical events with modified time-to-collision ≤ 3 s and called traffic conflicts. Note that interactions with modified time-to-collision greater than 3 s were not considered for analysis. This step ensures that the distribution’s right tail is selected where extreme events are present and used for crash risk estimation. This study focusses on rear-end conflicts characterised by a modified time-to-collision measure; however, the proposed framework can be applied to estimate any type of crash.

### Extracting covariates for the extreme value theory model

This study uses three datasets for crash risk estimation, including trajectories, traffic conflicts obtained from the automated video analysis platform, and loop detector data (containing information on signal timing and phasing) obtained from the relevant road authorities. These three datasets are processed simultaneously to obtain signal cycle level covariates. For this purpose, this study adapts the automated covariate extraction algorithm^6^ for rear-end conflicts. At the core of this algorithm is a data fusion technique combining three datasets to extract covariates at the signal cycle level. Using one category of Dasarathy’s classification for data fusion, that is, data in-feature out^30^, raw (or processed) data from multiple sources have been combined to extract features relevant to describing the process (i.e., rear-end crash risk at the signal cycle level).

The algorithm starts by identifying the timestamp of each signal cycle in the trajectory and conflict datasets. This step assists in assigning each trajectory to a corresponding cycle number in which it was recorded. Then, cycle numbers were assigned to each conflict obtained directly from the video analysis platform. Along with cycle numbers, other relevant information, such as green phase, yellow phase, red phase, and signal phasing strategy, indicating which direction is receiving green at what time, were also assigned to conflicts and trajectories. This step, therefore, ensured that loop detector data were no longer needed for further steps. In the next step, signal cycles containing rear-end conflicts were identified and considered for further processing. From each signal cycle, several covariates were extracted that characterise rear-end crash risk. These covariates were traffic flow (number of vehicles per signal cycle on a given lane), shockwave area (the triangle area where the traffic flow state is II following the four traffic flow states theory^31^), platoon ratio (proportion of vehicles arriving during the green phase multiplied by the ratio of the signal cycle length to the effective green time of the subject movement^32^), and average speed of vehicles during the signal cycle. The descriptive statistics of these covariates are presented in Table 2.

**Table 2:** Descriptive statistics of covariates for this study.

Intersection	Indicator	Mean	SD	Minimum	Maximum	Data collection detail
Appleby–Stafford	MTTC (s)	1.665	0.646	0.133	4.655	Camera 1: View 1: 12/11/19–13/11/19 (6 am–6 pm) Camera 1: View 2: 14/11/19–15/12/19 (6 am–6 pm) Camera 2: View 1: 12/11/19–13/11/19 (6 am–6 pm) Camera 2: View 2: 14/11/19–15/12/19 (6 am–6 pm)
	Flow (veh/cycle)	7.577	2.449	1	18	
	Speed (m/s)	3.535	1.237	1.218	9.414	
	Shockwave area (km s)	3.079	1.450	0.010	9.136	
	Platoon ratio	2.149	0.630	0.092	4.842	
	Number of cycles	632				
	Number of conflicts	2984				
Beaudesert–Granard	MTTC (s)	1.553	0.622	0.711	4.510	Camera 1: View 1: 20/8/19–21/8/19 (6 am–6 pm) Camera 1: View 2: 22/8/19–23/8/19 (6 am–6 pm) Camera 2: View 1: 20/8/19–21/8/19 (6 am–6 pm) Camera 2: View 2: 22/8/19–23/8/19 (6 am–6 pm)
	Flow (veh/cycle)	8.904	6.818	1	17	
	Speed (m/s)	5.262	1.423	1.2028	10.157	
	Shockwave area (km s)	2.695	0.0574	0	7.2635	
	Platoon ratio	2.199	1.105	0.054	5.141	
	Number of cycles	531				
	Number of conflicts	3829				
Logan–Kessel	MTTC (s)	1.66	0.638	0.361	5.046	Camera 1: View 1: 20/8/19–21/8/19 (6 am–6 pm) Camera 1: View 2: 22/8/19–23/8/19 (6 am–6 pm) Camera 2: View 1: 20/8/19–21/8/19 (6 am–6 pm) Camera 2: View 2: 22/8/19–23/8/19 (6 am–6 pm)
	Flow (veh/cycle)	2.363	4.065	1	28	
	Speed (m/s)	3.709	1.009	1.150	8.440	
	Shockwave area (km.s)	2.965	1.462	0.027	7.556	
	Platoon ratio	2.352	0.863	0.057	4.939	
	Number of cycles	617				
	Number of conflicts	2772				

### Traffic conflict measure

Following a comparison performed by Arun et al.^33^, modified time-to-collision (MTTC) is selected as the measure to best capture rear-end conflicts. However, it is worth noting that the proposed framework is flexible and can be employed using any conflict measure. MTTC relaxes the constant speed assumption of conventional time-to-collision by using the acceleration of vehicles^34^. Mathematically, MTTC can be calculated as

8 $${MTTC}_{t}=\frac{{\Delta v}_{t}\pm \sqrt{{\Delta v}_{t}^{2}+2{\Delta a}_{t}({x}_{L,t}-{x}_{F,t}-{D}_{L})}}{{\Delta a}_{t}},$$

where ${\Delta v}_{t}={x}_{F,t}-{x}_{L,t}$ is the relative speed of the conflicting vehicles at time *t*, ${x}_{L,t}$ and ${x}_{F,t}$ are the positions of leading vehicle and following vehicle at time *t*, respectively; ${\Delta a}_{t}={a}_{F,t}-{a}_{L,t}$ is the relative acceleration of the conflicting vehicles at time *t*; ${D}_{L}$ is the length of the leading vehicle. Equation (8) can lead to either (a) two positive values or (b) one positive value and one negative value. In case of (a), the minimum positive value is considered as MTTC, whereas in the case of (b), the positive value is considered as MTTC. Negated MTTC is used for developing the model, which provides a simpler and intuitive explanation to understand crash occurrence as MTTC = 0 implies that two trajectories overlap each other and reflect a crash.

### Crash data

The Department of Transport and Main Roads, Queensland, provided the crash records for the selected study sites for the years 2015–2019. Crash data contain the time of a crash, its location, and collision type. To avoid any mismatch between obtained traffic conflicts and crashes, filters were applied to the crash data in which video data were captured. These filters selected only the rear-end crashes that occurred during weekdays (Tuesday–Friday), during the daytime (6 am–6 pm), and during fair weather conditions.

## Results

### Extreme value model results

Separate extreme value models for each intersection have been developed. Four variants of Bayesian extreme value models are considered in this study, including a model without covariates (Model-1: a stationary model), a model with covariates added to the location parameter (Model-2: a non-stationary model with parameterisation of the location parameter), a model with covariates added to the scale parameter (Model-3: a non-stationary model with parameterisation of the scale parameter), and a model with covariates added to both the scale and location parameters (Model-4: a non-stationary model with parameterisation of the scale and location parameters). Note these four variants of models were developed for each intersection, and in total, 12 models were estimated. Several combinations of covariates were tested for Model-4, and the best-performing model was selected from the pool of candidate models based on goodness-of-fit measures.

In the Bayesian framework, two separate chains for each model parameter with different initial values were used. A total of 50,000 iterations were set to run, from which the first 20,000 iterations were discarded as burn-in samples, and the posterior estimates were obtained from the remaining 30,000 iterations. The convergence of all the models was checked by calculating the Gelman–Rubin statistics of two chains of each parameter as well with the help of trace plots. The value of Gelman–Rubin statistics was close to 1.1, indicating model convergence, and the trace plots displayed both chains are well mixed with each other, providing further evidence of model convergence.

The deviance information criterion (DIC) values are used for comparing the four variants of the models estimated in this study. As an illustration, the DICs for Logan–Kessel Intersection for Models-1, 2, 3, and 4 are 13,210, 10,332, 12,100, and 11,099, respectively, indicating that all non-stationary models indicate a better goodness-of-fit compared to the stationarity model. Further, this comparison also reveals the relatively superior performance of Model-2, implying that a model with covariates in the location parameter outperforms its competing models. This finding implies that the model with covariates in the location parameter provides a theoretical underpinning by informing how close or far the zero modified time-to-collision point is on the curve relative to the mean^6^. Similar findings were obtained for the other two study intersections.

Table 3 indicates the model estimation summary for each intersection, and it can be observed that results for all intersections are consistent with each other, and as such, the interpretation of model parameters will hold true for all study intersections. Although the parsimonious model only contains four covariates, several other variables were tested in the model, such as the number of conflicts less than a certain threshold. These variables were excluded from the final model because they (a) were found to be not significant (assessed by Bayesian credible intervals), (b) did not improve the model fit, and (c) neither mean crash estimates nor confidence intervals improved.

**Table 3:** GEV model estimation summary.

GEV parameter	Covariate	Appleby–Stafford		Beaudesert–Granard		Logan–Kessels	
		Estimate	SD	Estimate	SD	Estimate	SD
Location	Intercept	1.7507	0.0079	1.953	0.0658	1.429	0.1015
	Flow	0.00041	0.00001	0.0014	0.003	0.0045	0.0013
	Speed	0.0585	0.001	0.0096	0.0102	0.0279	0.0194
	Shockwave area	0.0323	0.009	0.2847	0.2495	0.0035	0.0131
	Platoon ratio	− 0.0036	0.0001	− 0.0036	0.0005	− 0.0159	0.0031
Scale	–	0.9601	0.0301	0.605	0.0421	0.7297	0.0326
Shape	–	0.045	0.0091	0.1732	0.0428	0.0228	0.0066
Deviance information criterion		10,558		11,332		10,332	
Observed crashes for 5 years (CI)		6 (2.2, 13.06)		16 (9.12, 25.96)		9 (4.16, 17.08)	
Estimated crashes for 5 years (CI)		4.7 (0, 31.69)		10.35 (0, 30.86)		6 (0, 29.62)	

*SD* standard deviation.

All the model parameters have consistent and intuitive sign conventions. The traffic flow parameter, for example, shows a direct relation with crash risk, implying that as traffic volume increases, rear-end crash risk is likely to increase. Theoretically, an increase in the flow parameter increases the value of the location parameter, moving it to the right side of the mean toward zero modified time-to-collision, reflecting increased crash risk. This finding is also reported by Zheng and Sayed^5^ that an increase in traffic exposure leads to an increased likelihood of crash occurrence because more vehicles would want to clear the intersection in a given green cycle, and as such, they tend to follow closely, thereby increasing the chances of rear-end collisions. Similar to traffic flow, the parameter for the average speed of vehicles is also found to be positively associated with crash risk. Vehicles travelling at higher speeds are at more risk of applying sudden brakes in response to changes in traffic lights, thereby increasing the likelihood of rear-end collisions^35^. The shockwave area also shows a positive relationship with crash risk, which can be explained by the fact that when longer queues are formed as a result of bigger shockwaves, the likelihood of rear-end collisions increases, primarily because more drivers want to clear the intersection in the immediate green phase of the cycle by tailgating their leaders, which decreases safety margin. Unlike all other model parameters, platoon ratio is negatively associated with crash risk, indicating that a higher platoon ratio leads to lower rear-end crash risk. A higher platoon ratio reflects a higher number of vehicles arriving during the green time, which can clear the intersection with a lower probability of rear-end crash risk.

The expected crash frequencies estimated by the EVT models, shown in Table 3, are compared with the historical crash records (i.e., observed crashes). Both mean crash estimates and confidence intervals are compared. Mean estimated crashes for *n* years can be calculated as $N=\frac{\widetilde{T}}{T}RC$, where *N* is the expected number of crashes during the duration $\widetilde{T}$, *T* is the data collection duration, and RC is the risk of crash. If $\widetilde{T}$ = 5 years, then *N* reflects the expected number of crashes for 5 years. Confidence intervals for the estimated crashes are obtained using a simulation method^36^, which utilises the distributions of model parameters to calculate the quantile of the distribution. For calculating the confidence interval of the observed crashes, the 95% Poisson confidence interval^2^ of the observed crashes using the true mean annual crashes, $\lambda$, is computed as $\left[\lambda :\frac{1}{2n}{\chi }_{{2y}_{0},0.975}^{2}\le \lambda \le {\chi }_{{2(y}_{0}+1),0.025}^{2}\right]$, where *n* = 5 years, and ${y}_{0}$ indicates the total number of crashes in the 5-year period for the study site. It can be seen from Table 3 that the mean estimated crashes are relatively close to the observed crashes for each site, and the mean estimated crashes are also within the confidence interval of the observed crashes. Note that comparing estimated crashes with aggregated crashes is a common practice for assessing real-time extreme value models in the literature; however, this does not discount the fact that these models should be assessed at the signal cycle level. A worthwhile research direction would be validating the signal cycle level crash risk estimates with granular crash data for a larger set of transport locations.

**Figure 5:**
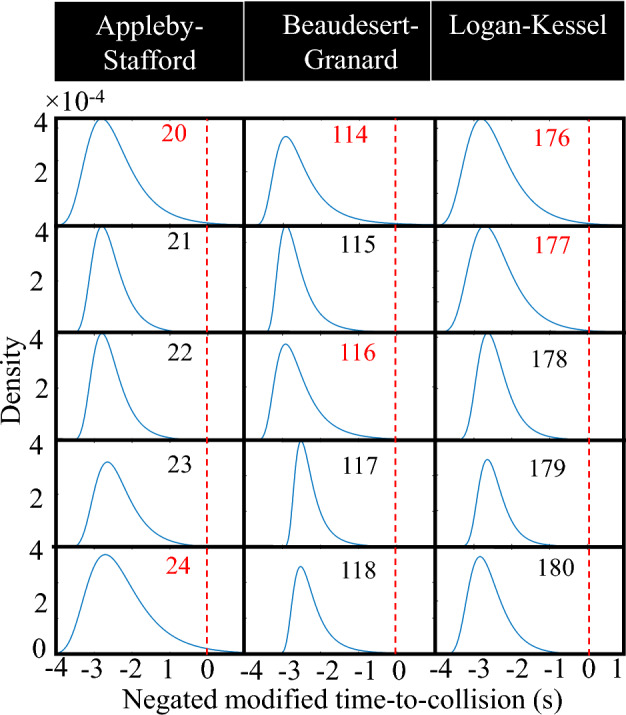
GEV distributions for some representative signal cycles.

The estimated non-stationary Bayesian models generate separate generalised extreme value (GEV) distributions corresponding to each signal cycle. As an illustration, a randomly selected set of five signal cycles from each intersection is presented in Fig. 5. Note that the numbers within each subfigure indicate the signal cycle number, and the signal cycle number in red indicates positive crash risk denoted by a red shaded area. The shape of the estimated GEV distribution provides insights into traffic conditions that lead to risky/safe cycles. More specifically, the tail of a GEV distribution ending after the negated modified time-to-collision (MTTC) = 0 indicates a positive crash risk. Figure 5 shows that two (out of five) signal cycles (20 and 24) for Appleby–Stafford road intersection have positive crash risk as their distributions have crossed the negated MTTC = 0. Similarly, two signal cycles are risky for Granard-Beaudesert Road and Logan–Kessel Road, as their distributions end after negated MTTC = 0. To complement this visual inspection, statistical analysis of GEV distributions is performed using a two-sample Kolmogorov–Smirnov test to compare the distributions across signal cycles. At a 95% confidence level, it is observed that cycles with positive crash risk are significantly different from other cycles (*p* value < 0.05), further confirming the difference in crash risk in different cycles.

From the fitted GEV distribution of each signal cycle, the crash risk of the corresponding signal cycle is obtained using Eq. (2). The signal cycle-level crash risks are used to develop and test the performance of the recurrent neural network model. Its results are described in the following subsection.

### Recurrent neural network model result

**Figure 6:**
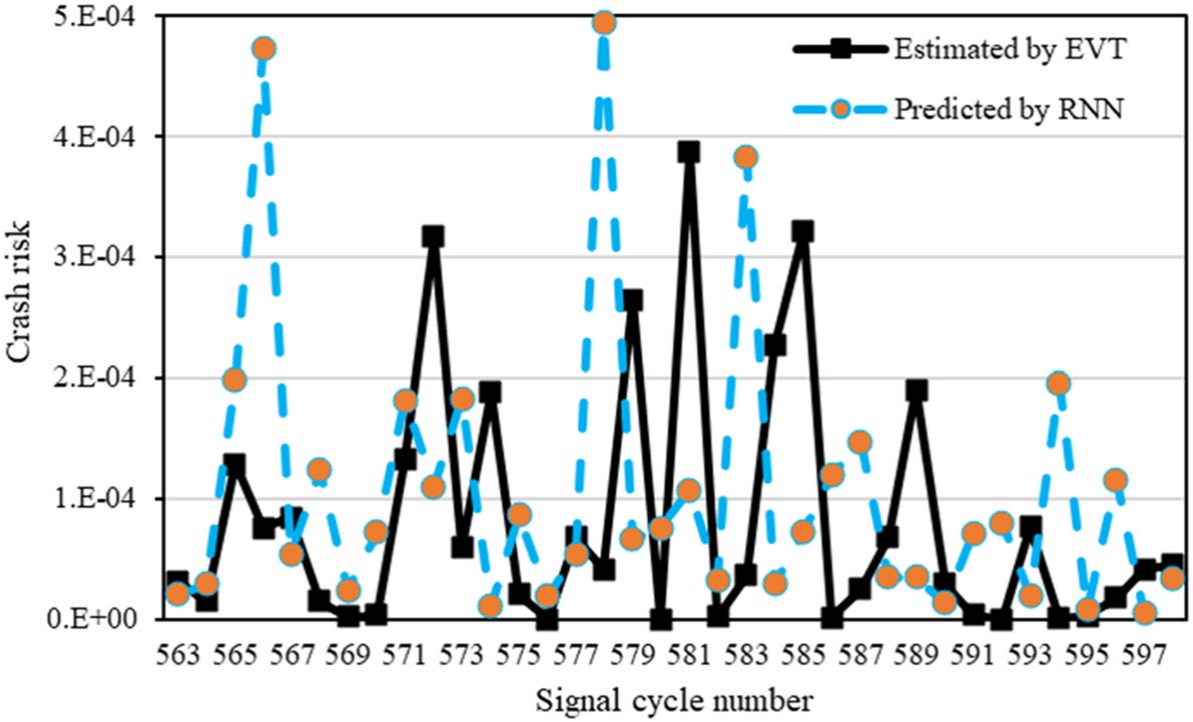
Crash risk prediction for Appleby–Stafford intersection.

The signal cycle-level crash risk obtained from the extreme value model is used as an input to the recurrent neural network (RNN) model to predict the crash risk for future signal cycles. Note that as this crash risk was obtained using signal-level covariates that influence the crash risk, we did not use them again for RNN modelling because they will only become redundant in the model. The RNN model is trained using 70% of the data in the framework of TensorFlow using ‘*Keras’* library in Python. Twenty percent of the remaining data has been used for model validation, whereas forecasting has been performed using the last 10% of data. Note that in the absence of ground truth for observed crash risk at the signal cycle level, the crash risk estimated from the GEV model is considered as the baseline for comparison with the crash risk predicted by the RNN model. To avoid overfitting, an early stopping technique has been applied during model training that monitors the validation loss and terminates the training process if the value of the error metric (here, mean absolute error) is not improved after some epochs. Note that the plot of training loss versus validation loss was used to examine overfitting, which revealed that both loss curves (training and validation) were close to each other when training was stopped, indicating no overfitting. Once the RNN model has been trained, and its convergence is ensured, the forecasting performance of the trained model is assessed. Two error metrics, namely mean absolute error (MAE) and relative absolute error (RAE, defined as the ratio of MAE and mean of crash risk estimated by EVT), are used to evaluate model performance, and results are presented in Table 4. Note that crash risk is predicted for each day of each intersection separately, and a typical graphical representation of estimated versus predicted crash risk for a day of the Appleby–Stafford intersection can be seen in Fig. 6.

**Table 4:** Prediction performance of the recurrent neural network model.

Intersection	Day	MAE	RAE
Logan–Kessel	1	1.22E−04	6.21E−02
	2	6.88E−04	1.09E−01
	3	2.73E−04	1.13E−01
	4	1.08E−03	1.08E−02
	Mean	5.41E−04	7.37E−02
Beaudesert–Granard	1	9.97E−05	1.76E−03
	2	1.25E−04	6.73E−03
	3	5.08E−05	1.06E−03
	4	8.99E−05	1.67E−03
	Mean	9.13E−05	2.81E−02
Appleby–Stafford	1	2.19E−04	5.71E−02
	2	1.04E−04	1.10E−03
	3	8.37E−05	9.29E−05
	4	7.65E−05	2.35E−04
	Mean	1.21E−04	1.46E−02

Table 4 indicates that the RNN model accurately predicts the crash risk for future cycles. For instance, the RAEs for Appleby–Stafford intersection for 4 days are 5.71E−02, 1.10E−03, 9.295E−05, and 2.35E−04, respectively, whereas the overall RAE for this intersection is 1.46E−02, which indicates that the model under/overpredicts the crash risk by 1.46%. Further, it can be noted from Table 4 that crash risk prediction error varies across sites, whereby the lowest overall error is observed for Beaudesert–Granard Intersection (RAE = 1.79E−02), and the highest overall error is found for Logan–Kessel Intersection (RAE = 7.37E−02).

Once the model performance is confirmed, the next natural step is to rigorously test this model and apply it to examine its performance in capturing day-wise crash risk variation, which is described in the ensuing paragraph.

## Discussion

### Performance comparison

In addition to the recurrent neural network (RNN) model, a long-short term memory (LSTM) model has been frequently used in the literature to model time-series data. As such, this study also developed LSTM models for crash risk prediction and compared their performance with the developed RNN model for all three signalised intersections. However, the comparison revealed that LSTM models did not outperform the RNN model for crash risk prediction, with a relatively higher mean absolute error. For instance, the overall mean absolute error of the LSTM model for the Appleby–Stafford intersection is 4.18E−04, and the corresponding error for the RNN model is 1.21E−04, which is more than three times lower than that of the LSTM model. Note that similar results were found for the other two intersections. LSTM networks are often applied and better suited for capturing long-term serial dependency, which in our case, implies that the crash risk of a future signal cycle is linked to the crash risk of several previous cycles. However, in reality, the crash risk of a future cycle is more likely to depend on the current signal cycle’s crash risk and perhaps on the crash risk of the immediate past signal cycle, indicating a short-term dependency, which could be one of the plausible reasons that the RNN model outperformed the LSTM model in this study. Although our finding contrasts with crash-based safety assessment studies^13,37^, some past studies confirm our finding^38^, reporting that when detecting the possibility of anomalous driving behaviour in routine tasks, an RNN model outperforms an LSTM model, which could be attributed to the short-term dependency of drivers’ past actions to their future actions.

Nevertheless, although the error of the LSTM model is relatively high, the trend predicted by the LSTM model is consistent with that of the RNN model, e.g., the highest and lowest errors are obtained for Logan–Kessel intersection and Beaudesert–Granard Intersection, respectively.

### Impact of train/test/forecast proportion on model performance

**Figure 7:**
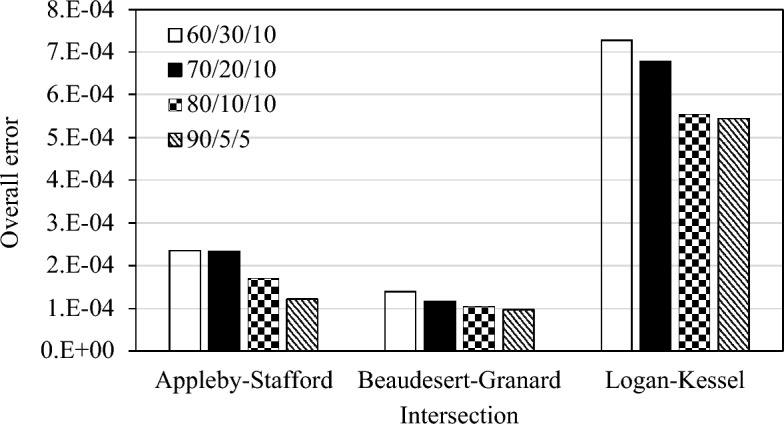
Performance variation with different train/test/forecast proportions.

The performance of the recurrent neural network model is further assessed for different data proportions. To this end, this study varies train/test/forecast proportion and evaluates the model performance for four combinations: 60/30/10, 70/20/10, 80/10/10, and 90/5/5, whereby the first number represents training data percentage (e.g., 60%, 70%, etc.), the second refers to the percentage of testing data (e.g., 30%, 20%, etc.), and the third number denotes the percentage of data used for out-of-sample forecasting. Figure 7 presents the comparison results in which the *y*-axis represents the average mean absolute error for 4 days for a given intersection. Irrespective of an intersection, it can be seen from this figure that the highest error is found when the 60/30/10 proportion of train/test/forecast data is used, whereas the lowest error is observed for the 90/5/5 proportion. This finding implies that when the model is trained with a sufficiently large training dataset, it is likely to yield a relatively smaller crash risk prediction error, implying that the model has learned the crash risk pattern well and is able to predict with good accuracy. Contrastingly, when the model is trained with the 60/30/10 proportion, more than one-third of the data is unseen to the model, and it is possible that the testing dataset contains some patterns that were not part of the training dataset, resulting in a high magnitude of prediction error. Although many studies use only one train/test data proportion (generally 70/30), neural network models (including recurrent neural networks) are reported to perform better when trained with a large training sample. For instance, Ng et al.^39^ reported the highest model performance when they used 90% of the data for training.

### Real-time forecasting outreach

**Figure 8:**
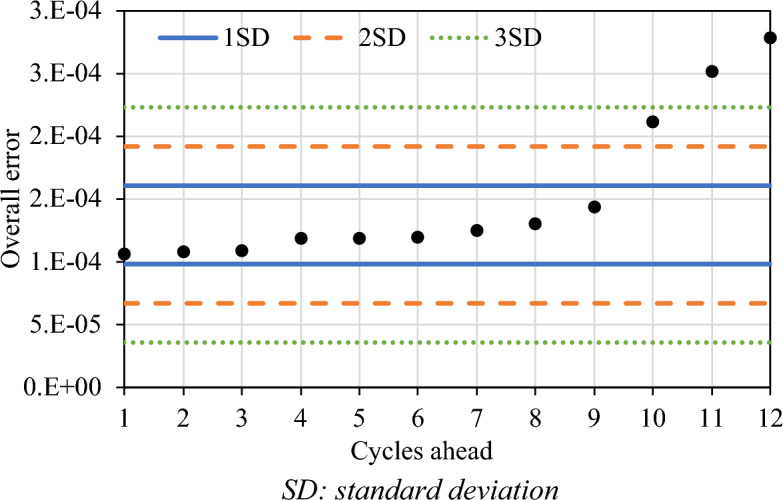
Forecasting outreach of the model.

Whilst the model performance has been assessed as a function of different train/test/forecast proportions in the previous subsection, it remains unclear how many signals cycles forecasting can be performed with acceptable accuracy. Knowledge of such forecasting capability will assist in evaluating safety in real time and implementing countermeasures. For this purpose, the overall forecasting error (average of mean absolute error of all 4 days for a given intersection) is calculated. As an example, Fig. 8 displays the performance of the recurrent neural model (RNN) for the Appleby–Stafford intersection and predictions for ten signal cycles can be seen. In this figure, three areas are denoted by the standard deviation of prediction error, whereby errors with two standard deviations represent a 95% confidence interval. It can be observed that the prediction error for nine signal cycles is within one standard deviation, whilst the prediction for the 10th cycle is outside two standard deviations, and the prediction of 11 or more cycles is outside three standard deviations. From this figure, it can be concluded that the developed RNN model can reasonably predict future crash risk. In this particular case, the RNN model predicts crash risk for nine future cycles, which is approximately about 20–25 min ahead. Note that although Fig. 8 and the aforementioned analysis are for Appleby–Stafford intersection, similar trends were observed for the other two intersections. Another key observation from Fig. 8 is the immediate future signal cycle exhibits lower prediction error, and the error magnifies when the prediction is performed for farther future cycles.

### Predicting crash risk for different periods of a day

An essential component of a real-time crash risk prediction model is the capability to accommodate for crash risk variations across different hours within a day and across days. As noted in a recent study^6^, crash risk varies across different periods of a typical day. The performance of the recurrent neural network (RNN) model has been compared in predicting the crash risk for nine future cycles for different periods of a day. Note that separate RNN models for peak hours of the day were developed using 70/20/10 proportion of data. As an illustration, Table 5 presents the model prediction results for Appleby–Stafford intersection, and it can be seen that the RNN shows a reasonable prediction error, with the lowest and highest errors observed for off-peak and morning (and evening) periods, respectively. It is interesting to note here that the prediction error remains similar across the 4 days in the off-peak period, which could be attributed to better capturing of crash risk that is often monotonous during the off-peak period. Contrastingly, the prediction error is relatively high when crash risk fluctuates more. To this end, more refinements in the current model are suggested.

**Table 5:** Prediction performance for different periods of the day for Appleby–Stafford intersection.

Day	Time periods of a day		
	Morning peak	Off-Peak	Evening peak
1	3.74E−04	1.75E−04	3.85E−04
2	2.33E−04	1.18E−04	3.91E−04
3	2.33E−04	1.83E−04	3.85E−04
4	1.89E−04	1.21E−04	2.97E−04

## Conclusions

This study proposed a bi-level real-time crash risk forecasting (RTCF) framework for signalised intersections. A Bayesian non-stationary block maxima model was developed and applied to rear-end conflicts in a signal cycle identified by a modified time-to-collision measure. Using the developed model, crash risk corresponding to each signal cycle was obtained, forming time-series data. To forecast the crash risk for future cycles based on the trend of crash risks of past and present signal cycles, the recurrent neural network model (RNN) was developed. The prediction accuracy of the RNN was assessed using two metrics, and the results showed a good performance accuracy in predicting the crash risk obtained from the Bayesian model. Further, a long-short term memory (LSTM) model was developed and compared with the RNN model, and the latter model outperformed the LSTM model, indicating the absence of long-term dependency of crash risk among a set of previous cycles. Further, for how long the RNN model can predict crash risk in future was examined, and it was found that the model can reasonably estimate the crash risk up to 20–25 min.

It is worth mentioning here that the proposed method also has the potential benefit as it does not require any new infrastructure to be installed but rather works on existing CCTV infrastructure. Further, the proposed method is also applicable for assessing the cost-effectiveness of engineering treatments, which are often based on crash data, whereas the current method does not rely on crash data to assess the effectiveness. For instance, very recently, Howlader et al.^40^ used extreme value modelling for before-after safety evaluation of part-time protected right-turn signals, and the proposed method was able to provide crash reduction rates.

As a cornerstone study in real-time crash risk prediction using the bi-level modelling framework, this study can be extended in several ways. First, determining the update frequency of model parameters is worth investigating an issue since the framework is expected to provide real-time insights as new data comes in. Second, combining data from multiple intersections to estimate a Bayesian model merits an investigation as previous research suggests the better performance of extreme value models when data are aggregated. Third, this study developed a simple recurrent neural network model for crash risk prediction, whose predictive capability can be significantly enhanced considering recent advancements in neural networks such as deep neural networks. As such, a comparison of the recurrent neural network model with deep learning and other baseline models warrants a future investigation. Fourth, given the high reliance on the crash-conflict relationship on trajectory data, it is necessary to understand how errors/noise in the data impact the model performance because it is often seen that data are contaminated with noise, such as freely available NGSIM datasets. Fifth, although the proposed framework is only tested for crash risk forecasting at isolated intersections, the proposed framework can be further leveraged to understand the spatial propagation of crash risk to multiple intersections and also upstream/downstream of a given intersection, which will require extensive video data coverage. Sixth, the current framework is only utilised for crash risk estimation and forecasting without delving into the aspect of traffic flow efficiency. As a follow-up study, examining the trade-off between safety and efficiency by applying the proposed real-time crash risk forecasting framework will be a worthwhile research direction. Seventh, developing a non-stationary extreme value model implicitly signifies the heterogeneous crash risk, stemming from different driving behaviours and traffic conditions. However, to explicitly account for driving style and consequently analyse its effects on real-time crash risk, additional data on driver characteristics could be collected to capture different driving styles. It is also worth investigating how econometric models for time-series forecasting, e.g., the autoregressive integrated moving average (ARIMA) model, perform for crash risk forecasting in comparison with the proposed RNN model. Finally, this study only developed a generalised extreme value model, whereas a generalised pareto model could also be investigated for real-time crash risk estimation.

## Data Availability

The datasets generated and/or analysed in this study are not publicly available, as raw video data are not allowed to be shared. We can only share the processed data, which is summarised in this paper.
